# Neurological Soft Signs, Spontaneous and Treatment Emergent Extrapyramidal Syndromes in Black Africans With First Episode Schizophrenia

**DOI:** 10.3389/fpsyt.2018.00172

**Published:** 2018-05-01

**Authors:** Akin Ojagbemi, Bonga Chiliza, Toyin Bello, Laila Asmal, Oluyomi Esan, Robin Emsley, Oye Gureje

**Affiliations:** ^1^Department of Psychiatry, World Health Organization (WHO) Collaborating Centre for Research and Training in Mental Health, Neurosciences, and Substance Abuse, University of Ibadan, Ibadan, Nigeria; ^2^Department of Psychiatry, Faculty of Medicine and Health Sciences, Stellenbosch University, Cape Town, South Africa

**Keywords:** neurological examination, side effects, neurodevelopmental defects, locomotor control, tardive dyskinesia, African ancestry

## Abstract

**Background:** Very little is known about the relationship between spontaneous and treatment-induced motor syndromes in Africans with first episode schizophrenia.

**Objective:** We investigated the association between spontaneous NSS and EPS, with treatment-induced EPS in a homogenous sample of Black Africans with first episode schizophrenia.

**Methods:** We examined Xhosa (South Africa) and Yoruba (Nigeria) patients, using the Neurological Evaluation Scale and extrapyramidal symptoms scale before and at 3 months after exposure to low dose flupenthixol decanoate. Pearson's correlations and Linear regression models, controlling for duration of untreated psychosis (D.U.P) and premorbid adjustments, were used in examining associations.

**Results:** Among 99 participants in the baseline sample, 91 (91.8%) and 20 (20.2%) had at least one definite NSS and EPS, respectively, before exposure to antipsychotics. Treatment-induced EPS were recorded in 34 (38.6%). Spontaneous EPS was associated with treatment-emergent Akathisia in participants with a longer D.U.P (*r* = 0.75, β = 0.70, *p* = 0.008). This association was specific for Parkinsonism (*r* = 0.75, β = 0.85, *p* = 0.008) and dyskinesia (*r* = 0.75, β = 1.70, *p* = 0.008).

**Conclusion:** Similar to previous findings for tardive dyskinesia in studies implementing longer-term follow-up, spontaneous EPS may also predict short-term antipsychotic-induced EPS such as akathisia. These results may be important for early identification of patients at risk of treatment-induced Akathisia-linked psychomotor agitation in first episode schizophrenia.

## Introduction

Early descriptions of schizophrenia include a broad spectrum of neurological examination abnormalities (1) such as Neurological soft signs (NSS) (2) and extrapyramidal syndromes (EPS) (3). Yet, the introduction of antipsychotics has led to a universal tendency to associate EPS with side effects (4) and NSS with neurodevelopmental defects (5).

In other perspective, NSS and EPS are two extremes in the diversity of neuromotor abnormalities in schizophrenia (6) [as NSS are subtle, but demonstrable, impairment in the ability to perform several motor and sensory tests on neurological examination 7, and EPS the more obvious abnormalities of locomotor control, involuntary movements, muscle tone, posture, and support 8). Spontaneous motor syndromes has emerged as a useful descriptor for signs (NSS and EPS) occurring before first exposure to antipsychotics (3, 9).

To date, the three cross-sectional studies of the relationship between NSS and treatment-emergent EPS in never-medicated first-episode schizophrenia have reported conflicting findings (10–12). However, a previous finding of an association between baseline NSS and subsequent development of tardive dyskinesia (TD) (13), across 24 months observation of a multiracial African sample is profoundly important for the prevention of EPS in susceptible patient populations. In this regard, some prior studies conducted in other multiracial samples suggest that persons of African ancestry may have a higher latent risk for motor syndromes (3, 14, 15). Yet, very little is known about the relationship between spontaneous and treatment-emergent motor syndromes in native (Black) Africans with first episode schizophrenia.

In the present study, which is based on a homogenous sample of Black Africans with first episode schizophrenia, we investigated the association between spontaneously occurring NSS and EPS, with 3 months treatment-induced EPS after exposure to minimum effective doses of flupenthixol decanoate.

## Methods

The present study is part of an investigation of the socio-demographic, clinical, biological, and treatment aspects of schizophrenia (and related disorders) in patients presenting for biomedical treatment for the first time as out- or in-patients in psychiatric hospitals and community clinics in Cape-Town South Africa and Ibadan Nigeria. It was conducted by multidisciplinary teams comprising of psychiatrists, mental health nurses, and social workers at the two locations, and funded by the New Partnership for Africa's Development through the Department of science and Technology of South Africa. Ethical approval was obtained from the University of Ibadan ethics committee and the Stellenbosch University Faculty of Medicine and Health Sciences Human Research Ethics Committees. Participants provided written, informed consent before interviews were conducted.

### Subjects

The subjects comprised anti-psychotic naïve or minimally treated patients with first episode schizophrenia or schizophreniform disorder (5 subjects had a lifetime exposure to oral anti-psychotics of less than 4 weeks). The diagnosis of the relevant disorder was made according to criteria in the Diagnostic and Statistical Manual for Mental Disorders-Fourth edition (DSM-IV) (16) by the attending psychiatrist at the study locations. Patients were eligible if they were aged between 16 and 45 years and have had less than 4 weeks of oral anti-psychotic exposure during their life time. All eligible patients were informed about the study, and the procedure was explained to them in their home language. We excluded patients with previous treatment of long acting depot antipsychotics, and those meeting DSM IV criteria for current substance abuse. Also excluded were patients with significant physical illnesses (e.g., chronic kidney disease) determined from the result of a full physical examination and appropriate laboratory investigations. Patients with mental retardation were excluded based on clinical history. Additional formal testing of cognitive capacity was not conducted. Participants comprised 81 Yoruba Nigerians, 18 Xhosa South-Africans. Participants did not receive monetary compensation for their participation. They were evaluated as far as possible before antipsychotic medications were prescribed. The following information was obtained from all patients: demographic data, personal history, psychiatric history, medical history, family history.

### Measures

The Structured Clinical Interview for the fourth edition of the Diagnostic and Statistical Manual of Mental Disorders (DSM-IV)- Patients edition (SCID-P) (17) was used in the recruitment of patients. The SCID-P provides for a standardized assessment that generates DSM-IV diagnoses using a semi-structured interview.

#### Assessment of neuromotor abnormalities

Tests assessing NSS and EPS (described below) were administered by independent raters (all psychiatrists) of African (AO, BC, and OE), and Asian (LA) descent after a 3 weeks training at the Tygerberg Hospital, Cape-Town, South Africa. Assessments were conducted in English and/or the home language of participants. The inter-rater reliability (IRR) conducted after training ranged from kappa = 0.62–0.82.

##### Neurological soft signs

NSS were evaluated using the Neurological Evaluation Scale (NES) (18). The NES is a well validated tool for neurological assessments in first episode schizophrenia. The scale includes “functionally meaningful” sub-scales which reflect signs of Motor co-ordination (tandem walk, rapid alternation, finger-to-thumb opposition, and finger-to-nose test), Sensory integration (audiovisual integration, stereognosis, graphesthesia, extinction, and right-to-left confusion tests), and Motor sequencing (first-ring, the first-edge-palm, Ozeretski, and rhythmic tapping tests). The other signs assessed by the NES include cerebral dominance, short-term memory, frontal release signs and eye movements. The NES items are scored with reference to the descriptive anchors provided on a three-point scale (no abnormality = 0; mild, but definite impairment = 1; marked impairment = 2) with the exception of “suck” and “snout” reflexes which are scored 0 or 2. In this study, a neurological abnormality was defined as the rating of 2 on any item (except cerebral dominance) on the NES. Each item was assessed according to a fixed order. For the present study, we considered the initial assessment of NSS before introduction of antipsychotics.

##### Extrapyramidal syndromes

EPS were sassed using the Extrapyramidal Symptom Rating Scale (ESRS) (19). The ESRS was designed to rate three main types of EPS: Parkinsonism, dystonia and dyskinetic symptoms. The measure also includes one question to cover akathisia. These categories of EPS are measured assessed in three components of the ESRS. A questionnaire and behavioral scale which includes subjective questions covering the categories of EPS as follows: Questions 1–5 and 7 (Parkinsonism), 6 (Akathisia), 8 (Dystonia), and 10–11 (Dyskinesia). The status of each EPS is rated as 0 (if absent), 1 (mild), 2 (moderate), and 3 (severe). The questionnaire component of the ESRS helps to differentiate subjective akinesia from depressive symptoms related to the anti-adrenaline effects of some antipsychotic drugs. But they also help to differentiate akathisia from anxiety and psychotic agitation. Also included in the ESRS are sections for physical examination covering Parkinsonism, dystonia, and dyskinetic movements as well as sections for clinical global impression items. The status of EPS on clinical examination is rated as 0 (if normal), 1 (very mild), 2 (mild), 3 (moderate), 4 (moderately severe), 5 (severe), 6 (extremely severe). In line with previous studies (20, 21), EPS was considered as present in this study where there was a rating suggesting mild but definite extrapyramidal sign or symptoms in the questionnaire scale (representing self-reported EPS) or clinical examination (representing observed EPS) components of the ESRS. The ESRS was administered before exposure to antipsychotics, followed by 2 weekly ESRS assessments throughout the duration of the study.

#### Psychiatric assessment

The severity of the baseline psychopathology was evaluated by administering the Positive and Negative Syndrome Scale (PANSS) (22). The model of the PANSS adopted in this study (23) included five factor analysis derived domain for “positive symptoms” (delusions, hallucinations, unusual thought content, suspiciousness, and grandiosity), “negative symptoms” (lack of spontaneity, blunted affect, emotional withdrawals, apathetic social withdrawals, motor retardation, poor rapport and active social avoidance), “disorganization” (stereotyped thinking, poor attention, disorientation, conceptual disorganization and difficulty in abstraction), “excitement/hostility” (poor impulse control, excitement, hostility, and uncooperativeness), and “emotional distress” (anxiety, depression, guilt, and tension). The PANSS five factor solution (23) was adopted for this study based on its stability and superior validity when compared to the older three factor model (22).

The overall clinical status was assessed using the Clinical Global Impression (CGI-Severity) (24), while pre-morbid adjustment was explored using the Pre-morbid Adjustment Scale (PAS) (25). These measures have been used for the assessments of African patients with schizophrenia in previous studies (26).

Duration of untreated psychosis (D.U.P) was defined as the period in months from the onset of psychotic phenomena to first presentation for biomedical treatment. Onset of psychosis was defined as the presence for 1 week or more of psychotic symptoms with marked deterioration of functioning.

In all cases, pre-morbid functioning was the retrospective rating of patients' functioning up to 6 months before the defined onset of psychosis.

### Treatment

After ruling out hypersensitivity by administering 1 milligram (mg) of oral flupenthixol after the first assessment of clinical and outcome characteristics, flexible doses of deep intramuscular flupenthixol injections starting from 5 or 10 mg in 2 or 4 weekly intervals were administered, with increases by 5 mg for up to a maximum of 30 mg depending on age, tolerability or response. Depot antipsychotic was chosen for this study to rule out covert non-adherence, a common phenomenon in first episode schizophrenia (27).

### Statistical analysis

Descriptive statistics such as mean, median and standard deviations were used to summarize quantitative variables, while frequencies and proportions were used for discrete variables. Spontaneous motor syndromes were those occurring before first exposure to flupenthixol decanoate (28). Emergent EPS was determined after exposure to flupenthixol decanoate and censoring participants meeting criteria for spontaneous EPS. The frequency and severity of spontaneous motor syndromes (NSS and EPS) are presented along with their subcategories.

For the relationship between spontaneous motor syndromes and treatment-emergent EPS, we first conducted a Pearson correlation between spontaneous NSS and EPS total scores with the scores for treatment-emergent EPS. Where significant correlation coefficients were observed, the relevant spontaneous motor syndrome scores (NSS or EPS) were entered as independent variables into separate linear regression models. Given previous observations that the frequency and severity of motor syndromes in schizophrenia may be affected by both premorbid adjustments and D.U.P (20), we conducted sub-group analyses controlling for types of D.U.P (</≥12 months) and premorbid adjustment (</≥mean for the entire sample).

Analyses were conducted using Stata version 13.0 (29). Values of *p* < 0.05 were considered significant.

## Results

**Figure 1:**
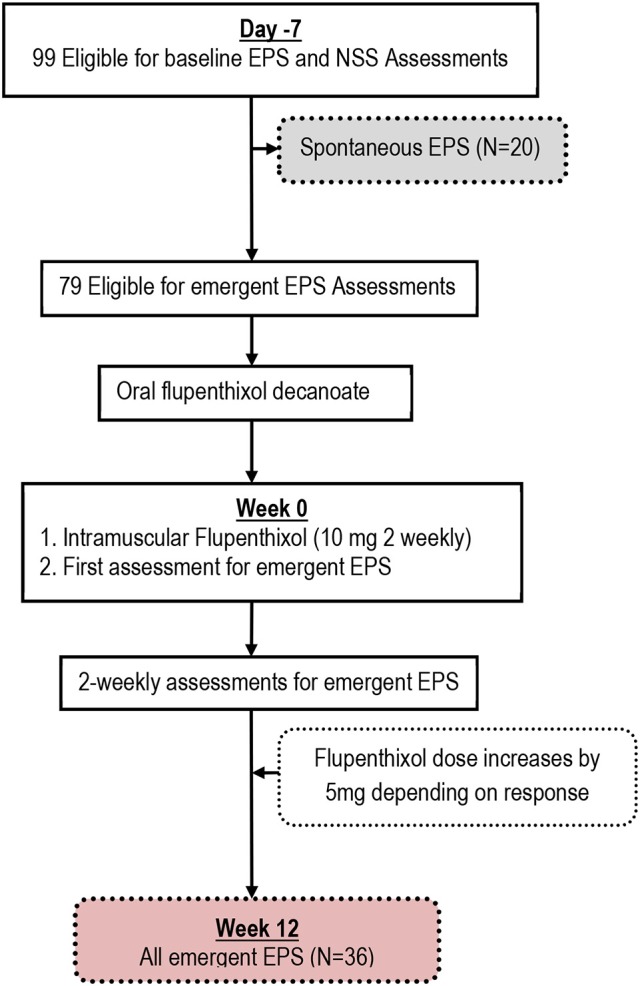
Flow chart showing Spontaneous and Emergent Extrapyramidal Syndromes (EPS) in the study of Black Africans with first episode schizophrenia.

A total of 99 participants met study inclusion criteria and underwent full baseline assessments (Figure 1). Table 1 shows the baseline characteristics of the study sample. Never married and unemployed young men, who were between the ages of 18 and 32 years at onset of psychosis, predominated in the sample. They were mostly markedly ill with prominent positive and disorganization psychopathologies. The median D.U.P was approximately 2.5 years (Table 1).

**Table 1:** Characteristics of 99 Black African patients with first episode schizophrenia.

**Characteristics**	**%**
**GENDER**	
Male	58.6
Female	41.4
**MARITAL STATUS**	
Never married	74.7
Ever married	25.3
**FAMILY HISTORY OF PSYCHOSES**	
No	84.7
Yes	15.3
**EMPLOYMENT**	
Unemployed	88.9
Employed	11.1
**DUP (MONTHS)**	
< 12	51.5
≥12	48.5
**PSYCHOPATHOLOGY (PANSS)**	
Positive	47.5
Negative	37.4
Disorganized	46.5
Excitement/hostility	16.2
Emotional distress	8.1
	**Mean (SD)**
Age at onset of Psychosis	25.5 (6.9)
Age at presentation	27.9 (6.6)
D.U.P in months, median	28.8 (46.5) 11.0
Neurological soft signs	18.0 (11.1)
Motor coordination	2.4 (2.1)
Sensory integration	4.7 (3.1)
Motor sequencing	5.2 (2.9)
Childhood premorbid adjustment	1.9 (2.8)/3.3 (2.7)
Adolescence premorbid adjustment	3.7(3.9)/3.4 (2.6)
Total PANSS scores	76.8 (18.5)
CGI-severity	5.1 (0.9)
Functioning (SOFAS)	44.6 (13.1)

*D.U.P, Duration of untreated psychoses; P.A.S, Pre-morbid adjustment scale; PANSS, Positive and Negative Syndrome Scale; CGI, Clinical Global impression; SOFAS, Social and Occupational functioning scale*.

Table 2 describes the frequency and severity of spontaneous motor syndromes and their relationship with 3-months treatment-emergent EPS. About 91.8 and 20.2% of participants had at least one definite spontaneous NSS and EPS respectively. The only spontaneous motor syndrome not recorded in the sample was dystonia. Newly emergent EPS was recorded in 34 (38.6%) participants within 3 months of exposure to antipsychotics. Whereas the overall domain scores of spontaneous motor syndromes were not associated with treatment emergent EPS, spontaneous dyskinesia was significantly associated with treatment emergent akathisia (*r* = 0.55, β = 1.65, *p* = 0.008). Table 2 also presents the distribution of doses of flupenthixol decanoate. Antipsychotic dose was not associated with treatment emergent motor syndromes.

**Table 2:** Association of spontaneous motor syndromes with 3-months treatment induced extrapyramidal signs (EPS) assessed using the Extrapyramidal Symptom Rating Scale (ESRS) after exposure to flupenthixol decanoate in Black Africans with first-episode schizophrenia.

**Spontaneous motor syndromes (***N*** = 99)**			**3-months treatment-emergent Extrapyramidal signs (Pearson coefficients/βeta for linear regression)^a^**				
**Neurological soft signs**	**%**	**Mean (*SD*)**	**Parkinsonism**	**Akathisia**	**Dyskinesia**	**Dystonia**	**Overall**
Sensory integration	45.9	2.4 (2.1)	0.11	0.20	0.36	0.05	0.13
Motor coordination	73.5	4.7 (3.1)	0.04	0.17	0.41	0.19	0.03
Motor sequencing	78.6	5.2 (2.9)	0.03	0.01	0.03	0.04	0.03
Total NES	91.8	18.0 (11.1)	0.06	0.17	0.09	0.11	0.07
**EXTRAPYRAMIDAL SIGNS**							
Parkinsonism	18.2	0.71 (2.36)	^b^	0.03	0.61	0.09	^b^
Akathisia	3.0	0.03 (0.17)	^b^	^b^	^b^	0.03	^b^
Dyskinesia	3.0	0.10 (0.68)	^b^	0.55^*^/1.65^*^	^b^	^b^	^b^
Dystonia	^b^	^b^	^b^	^b^	^b^	^b^	^b^
Overall	20.2	0.84 (2.46)	^b^	0.13	0.61	0.09	^b^
**Flupenthixol decanoate**	**Minimun/Maximum**	**Mean (***SD***)**					
Dose	5 mg/30 mg	10.3 (2.27)	0.12	^b^	^b^	^b^	0.12

*1. Participants were considered as having definite EPS in this study when they have a rating of ≥2 on the ESRS, 2. Percentages are based on the entire sample (N = 99)*,

a *Participants had definite EPS only after exposure to flupenthixol decanoate (i.e., had a score of < 2 on the ESRS before exposure to treatment)*,

b *Participants with treatment-emergent EPS but who had a score of zero on the ESRS before exposure to flupenthixol decanoate*,

* *p < 0.05*.

The results of analyses controlling for D.U.P and premorbid adjustment is presented in Table 3. Spontaneous EPS was associated with treatment-emergent Akathisia in participants with a longer D.U.P (*r* = 0.75, β = 0.70, *p* = 0.008). This association was specific for Parkinsonism and dyskinesia (Table 3). Conversely, spontaneous motor sequencing NSS was associated with treatment-emergent dystonia (*r* = −0.99, β = 1.89, *p* = 0.014).

**Table 3:** Relationship of spontaneous and 3-months treatment induced motor syndromes adjusted by duration of untreated psychosis and Premorbid adjustments in Black Africans with first-episode schizophrenia.

**Spontaneous motor syndromes**	**3-months treatment-emergent Extrapyramidal signs (Pearson coefficients/βeta for linear regression)^a^**									
	**Duration of untreated Psychosis <12 months**					**Duration of untreated Psychosis ≥12 months**				
**Neurological soft signs**	**Parkinsonism**	**Akathisia**	**Dyskinesia**	**Dystonia**	**Overall**	**Parkinsonism**	**Akathisia**	**Dyskinesia**	**Dystonia**	**Overall**
Sensory integration	0.11	0.62	0.95	0.93	0.11	0.06	0.07	^b^	0.37	0.05
Motor coordination	0.35	0.58	0.00	0.36	0.39	0.26	0.11	^b^	0.18	0.28
Motor sequencing	0.41	0.16	0.72	0.93	0.40	0.28	0.18	^b^	0.33	0.30
Total NES	0.35	0.43	0.73	0.97	0.39	0.13	0.06	^b^	0.26	0.16
**EXTRAPYRAMIDAL SIGNS**										
Parkinsonism	^b^	0.29	0.50	0.27	^b^	^b^	0.75^*^/0.85^*^	^b^	0.10	^b^
Akathisia	^b^	^b^	^b^	^b^	^b^	^b^	^b^	^b^	0.61	^b^
Dyskinesia	^b^	^b^	^b^	^b^	^b^	^b^	0.75^*^/1.70^*^	^b^	^b^	^b^
Dystonia	^b^	^b^	^b^	^b^	^b^	^b^	^b^	^b^	^b^	^b^
Overall	^b^	0.29	0.50	0.27	^b^	^b^	0.75^*^/0.57^*^	^b^	0.00	^b^
	**Good premorbid adjustment**					**Poor premorbid adjustment**				
Sensory integration	0.21	0.08	^b^	0.80	0.23	0.01	0.15	0.36	0.80	0.02
Motor coordination	0.24	0.23	^b^	0.94	0.23	0.26	0.42	0.41	0.45	0.29
Motor sequencing	0.37	0.14	^b^	0.99^*^/1.89^*^	0.33	0.15	0.39	0.03	0.82	0.17
Total NES	0.22	0.23	0.09	0.90	0.23	0.07	0.24	0.09	0.74	0.09
**EXTRAPYRAMIDAL SIGNS**										
Parkinsonism	^b^	0.34	^b^	0.08	^b^	^b^	0.15	0.61	0.24	^b^
Akathisia	^b^	^b^	^b^	^b^	^b^	^b^	^b^	^b^	0.58	^b^
Dyskinesia	^b^	0.56	^b^	^b^	^b^	^b^	^b^	^b^	^b^	^b^
Dystonia	^b^	^b^	^b^	^b^	^b^	^b^	^b^	^b^	^b^	^b^
Overall	^b^	0.42	^b^	0.08	^b^	^b^	0.15	0.61	0.11	^b^

*1. Participants have poor premorbid adjustment when their score on the premorbid adjustment scale is less than the mean score for the entire sample, 2. Participants were considered as having definite EPS in this study when they have a rating of ≥2 on the ESRS (suggesting definite EPS), 3. Percentages are based on the number entire sample (N = 99)*,

a *Participants who record definite EPS for the first time after exposure to flupenthixol decanoate*,

b *Participants with treatment emergent EPS but who had a score of zero on the ESRS before exposure to flupenthixol decanoate*,

* *p < 0.05*.

## Discussion

The present study is based on a homogenous sample of native (Black) Africans with first episode schizophrenia or schizophreniform disorder. Our main finding was that spontaneous EPS was associated with treatment emergent akathisia. This relationship was especially evident when considering spontaneous dyskinesia and Parkinsonism in patients with a longer D.UP. We also found an association between spontaneous motor sequencing NSS and treatment-emergent dystonia in patients with better premorbid functioning.

Our finding suggesting that spontaneous motor syndromes may predict treatment-emergent EPS are largely in keeping with previous studies (10–12). Whereas we found that spontaneous EPS is associated with treatment-emergent Akathisia, prior studies implementing a longer term follow-up of up to 24 months have demonstrated this relationship for treatment-emergent dyskinesia (13). Compared with dyskinesia, akathisia is an earlier onset antipsychotic induced EPS (30). We have implemented a short-term (3-months) follow-up in the present study. This relatively short period of antipsychotic exposure may have been insufficient to demonstrate large increases in dyskinesia in our sample. For example, we found only 5 participants with treatment-emergent dyskinesia the present study. This relatively small number may have been insufficient to power a significant relationship for dyskinesia even if it exists in our sample. Even so, our finding with respect to akathisia is important as it shows that, as with dyskinesia, spontaneous EPS may also predict the emergence of earlier onset antipsychotic-induced EPS. This finding may also be of value for the prevention of akathisia-linked psychomotor agitation in clinical samples through the early identification of susceptible patients. A future longer-term follow-up of our sample will be needed to investigate whether as in other populations, spontaneous motor syndromes predict treatment emergent dyskinesia in Black Africans with first episode schizophrenia.

Our finding of an association between spontaneous motor sequencing NSS and treatment-emergent dystonia in patients with better premorbid functioning was unexpected and must be interpreted with caution. However, it is feasible that such unpredictable findings suggest that there are more complex relationships between the diverse neuromotor abnormalities in schizophrenia. For example, it is plausible that spontaneous and emergent motor syndromes represent distinct, and potentially different, aspects of schizophrenia. This proposition is in keeping with the findings of distinct structural and functional changes (in the basal ganglia, pre-frontal and temporal cortices, as well as the cerebellum) in relation to the clinical spectrum of neuromotor abnormalities in schizophrenia (31, 32). Even so, the aforesaid association between spontaneous motor sequencing NSS and treatment-emergent dystonia in patients with better premorbid functioning may well have occurred by chance.

### Strengths and limitations

This study is based on a homogenous sample of Black Africans with first episode schizophrenia. Given previous observations that race may be important in determine putative risk for neuromotor abnormalities in schizophrenia (14, 15), the evidence provided by the present study is important for better understanding of the nature of neuromotor disturbances of schizophrenia. Prior information on neuromotor abnormalities in schizophrenia, including their evolution in relation to treatment has been based on mostly Caucasian or Mixed population (10, 12, 21). A key limitation of the present study is that the ESRS, which we have used for the ascertainment of EPS, does not provide a robust evaluation of all EPS and is more weighted toward Parkinsonism compared with, for example, Akathisia.

## Conclusion

Spontaneous EPS is predictive of treatment-emergent akathisia during a relatively short period of follow-up. The findings overlap with those of a previous 24 months longitudinal observation in a mixed population of Africans where, given the long period of observation, the relationship between spontaneous motor syndrome and treatment-induced dyskinesia was discernible. The result of the present study is of value for prevention of akathisia-linked psychomotor agitation in clinical samples through the early identification of susceptible patients. A future longer-term follow-up of our homogenous sample of Black Africans with first episode schizophrenia will investigate whether as in other populations, spontaneous motor syndromes predict treatment emergent dyskinesia in this population.

## Author contributions

All authors listed have made a substantial, direct and intellectual contribution to the work, and approved it for publication.

### Conflict of interest statement

The authors declare that the research was conducted in the absence of any commercial or financial relationships that could be construed as a potential conflict of interest.
